# Ethylene-*co*-norbornene Copolymerization Using a Dual Catalyst System in the Presence of a Chain Transfer Agent

**DOI:** 10.3390/polym11030554

**Published:** 2019-03-22

**Authors:** Laura Boggioni, Diego Sidari, Simona Losio, Udo M. Stehling, Finizia Auriemma, Anna Malafronte, Rocco Di Girolamo, Claudio De Rosa, Incoronata Tritto

**Affiliations:** 1 Istituto per lo Studio delle Macromolecole (ISMAC), Consiglio Nazionale delle Ricerche (CNR), Via E. Bassini 15, 20133 Milano, Italy; boggioni@ismac.cnr.it (L.B.); sidari@ismac.cnr.it (D.S.); s.losio@ismac.cnr.it (S.L.); 2 TOPAS Advanced Polymers GmbH, Paulistrasse 3, 65926 Frankfurt am Main, Germany; udo.stehling@gmx.net; 3 Dipartimento di Scienze Chimiche, Università di Napoli Federico II, Complesso Monte S. Angelo, Via Cintia, 80126 Napoli, Italy; malafronte@unina.it (A.M.); digirolamo@unina.it (R.D.G.); claudio.derosa@unina.it (C.D.R.)

**Keywords:** cycloolefin copolymerization, metallocenes, ZnEt_2_, segmented polymers, mechanical properties

## Abstract

Ethylene-*co*-norbornene copolymers were synthesized by a dual catalyst system at three concentrations of norbornene in the feed and variable amounts of ZnEt_2_, as a possible chain transfer agent. The dual catalyst system consists of two *ansa*-metallocenes, isopropyliden(η^5-^cyclopentadienyl)(η^5^-indenyl)zirconium dichloride (**1**) and isopropyliden(η^5^-3-methylcyclopentadienyl)(η^5^-fluorenyl)zirconium dichloride (**2**), activated with dimethylanilinium tetrakis(pentafluorophenyl)borate, in presence of TIBA. Values of norbornene content, molecular mass, glass transition temperature, and reactivity ratios *r*_11_ and *r*_21_ of copolymers prepared in the presence of **1**+**2** are intermediate between those of reference copolymers. The study of tensile and elastic properties of ethylene-*co*-norbornene copolymers (poly(E-*co*-N)s) gave evidence that copolymers were obtained in part through transfer of polymer chains between different transition metal sites. Mechanical properties are clearly different from those expected from a blend of the parent samples and reveal that copolymers obtained in the presence of **1**+**2** and ZnEt_2_ consist of a reactor blend of segmented chains produced by exchange from **2** to **1** and **1** to 2 acting as the ideal compatibilizer of chains produced by the chain transfer from **1** to **1**, and from **2** to **2**.

## 1. Introduction

Progress in polymerization catalysis has permitted the synthesis of olefin block copolymers. Tailored block copolymers are synthesized by sequential synthesis block by block through living polymerization catalysts [1,2,3]. They often provide materials whose mechanical properties are remarkable in comparison with those of homopolymer blends or random copolymers with the same chemical composition, because of microphase separation of the different blocks [1,2,4]. Living single-site olefin polymerization catalysts do exist. In a living polymerization system, there is no chain termination, thus only one chain can be grown per metal center of quite expensive catalysts [5,6]. Likely due to high costs of living polymerization methods and poor processability of the monodisperse polymers, these materials have found limited commercial application.

Coordinative chain transfer polymerization (CCTP), where reversible transfer of the growing macromolecular chain occurs from the active catalytic species to the chain transfer agent (CTA), typically a metal alkyl compound, such as zinc alkyl, has been developed [7,8,9,10,11,12]. This strategy enables one to obtain the growth of several polymer chains per catalyst, reducing the amount of expensive transition metal-based catalysts, and offering control of the molecular weight of the polymer.

**Scheme 1:**
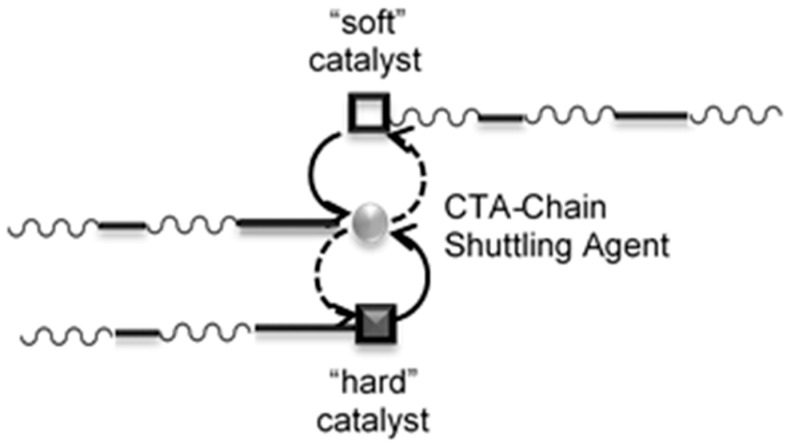
Chain shuttling of a growing polymer chain among different active catalyst sites, mediated by a chain transfer agent (CTA).

Recently, the chain-shuttling copolymerization of two different comonomers by two Group 4 metal catalysts, with different monomer selectivity, has been developed as a powerful methodology for the synthesis of olefin block copolymers with different segments (Scheme 1) [13,14,15]. The multi-block copolymers obtained (MBCPs) represent a great advance in polyolefin synthesis, which allows one to achieve polymer properties previously inaccessible to olefin-based materials. The success of chain-shuttling copolymerization reported to date mainly regards the copolymerization of ethylene and propylene (or 1-hexene) [13,14,15,16] as well as of isoprene and styrene [17,18]. This is probably due to the difficulties in finding a good combination of catalysts and chain shuttling agents. Interest in understanding polymerization mechanism of this catalysis [19,20,21] and properties of polymers obtained by this technology [22,23,24] is revealed by recent excellent studies. Seldom one of the catalysts is an *ansa*-metallocene.

Probably for these reasons, chain-shuttling copolymerization of norbornene (N) and ethylene (E), made possible by the discovery of metallocene catalysis by Kaminsky [25,26,27,28,29,30,31], has remained unexplored to date. For example, while there are significant studies on chain-growth termination and chain-transfer reactions for homo- and copolymerizations of α-olefins with transition metal catalysts, only one study on the exchange of ethylene-*co*-norbornene polymer chain between ansa-zirconocene catalysts and aluminum or zinc alkyls was found in the literature [32] before our recent studies [33].

Ethylene and norbornene copolymers, poly(E-*co*-N)s, are one class of polymers made possible by the discovery of metallocene catalysis [25,26,27,28,29,30,31]. Random poly(E-*co*-N)s with norbornene content above 20 mol % are amorphous materials with an exceptional combination of properties [16,17,18,19,20,21,22]. They are materials of high commercial interest used in optics, capacitor films, medical and diagnostic applications food and drug packaging. They have a moisture barrier 4–5 times higher than LDPE, high glass transition temperature, thermoformability, transparency, stiffness and chemical inertness; in addition, these copolymers can be easily recycled. Owing to their value generating characteristics, cyclic olefin copolymers are one of few examples of copolymers industrially produced by two industries, TOPAS and Mitsui, with tradenames TOPAS [34,35] and APEL [36], respectively. The main characteristics of these materials are directly linked to norbornene content, norbornene distribution and copolymer microstructure (e.g., isolated ENE units, alternating NEN units or presence of NN dyads, and NNN blocks) and sequence stereoregularity.

**Figure 1:**
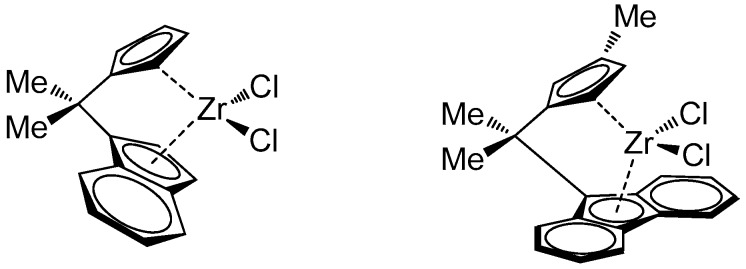
*Ansa*—zirconocene based precursors studied.

To expand physical properties of poly(E-*co*-N)s with high N content, which have high *T*_g_s but are rather brittle, and to widen their applications, we recently explored the effect of ZnEt_2_ as a potential chain transfer agent on the ethylene-norbornene copolymerization [33], by means of two *C*_1_-symmetric *ansa*-metallocenes isopropyliden(η^5^-cyclopentadienyl)(η^5^-indenyl)zirconium dichloride (**1**) and isopropyliden(η^5^-3-methylcyclopentadienyl)(η^5^-fluorenyl)zirconium dichloride (**2**), (Figure 1). They produce poly(E-*co*-N)s with different microstructures and molar masses. Random poly(E-*co*-N)s with lower molar masses and highly alternating isotactic poly(E-*co*-N)s with high molar mass are obtained with **1** and **2**, respectively [32,33,37,38,39,40]. Ethylene-*co*-norbornene copolymerization experiments were conducted using **1** and **2**, separately, activated with dimethylanilinium tetrakis(pentafluorophenyl)borate in the presence of variable amounts of diethyl zinc as chain transfer agent, at different [N]/[E] feed ratios [33]. These investigations revealed that chain transfer to zinc alkyl occurs in all copolymerization reactions, in presence of diethyl zinc, along with the typical chain transfers of E-*co*-N polymerization. Chain transfer of ethylene-norbornene polymer chains to ZnEt_2_ is more efficient in copolymers at low N content and is more probable when the last inserted unit is ethylene because of lower steric constraints. The two catalysts show different behavior in copolymerization reactions at high N content. The analysis of mechanical properties showed that CTA influences the polymer chain structure. An increase of CTA changed ductility, rigidity, and mechanical strength. Thus, although the catalytic systems do not behave as a pure CCTP, it was demonstrated that chains produced when adding ZnEt_2_ are, at least in part, characterized by segments made after exchange between different metal centers, possibly jointed through ethylene units.

In this work, the effect of variable amounts of ZnEt_2_ as a potential chain-transfer agent on poly(E-*co*-N)s, synthesized in the presence of a dual catalyst system composed of two different ansa metallocene catalysts **1** and **2**, was studied. The aim was to verify if polymer chain transfer between different transition metal sites can take place and modify polymer properties. Polymer microstructure and comonomer content were obtained by ^13^C-NMR. First- and second-order statistical models were used to fit the experimental tetrad distributions of copolymers. Molecular weight (*M*_w_) and molecular weight distribution (by SEC), the structural and the thermal properties (by diffraction analysis and DSC, respectively) and the mechanical and elastic properties were determined.

Based on the analysis of polymer microstructure, molar masses, structure, thermal and mechanical behavior provided evidences that poly(E-*co*-N) chains contain multi-blocks formed via exchange of polymer chains between different transition metal sites and that the obtained copolymer materials have unique properties due to segmented chains produced by exchange from **2** to **1** and **1** to **2** acting as the ideal compatibilizer.

## 2. Materials and Methods

### 2.1. Materials

All experiments were performed under nitrogen in a glove box or by classical Schlenk-line techniques. Nitrogen and ethylene gases were purified by passage through BTS catalyst, molecular sieves and CaCl_2_. Toluene used as reaction solvent was purified by distillation from Na prior use. C_2_D_2_Cl_4_ (99.9% of D), norbornene (98%), diethylzinc solution (10 wt % in toluene), and triisobutylaluminum (TIBA neat) were purchased from Sigma Aldrich (St. Louis, MO, USA). Norbornene was distilled from Na, and used as toluene stock solution. Diethylzinc solution was handled in a glove box. Metallocenes **1** (isopropyliden(η^5^-cyclopentadienyl)(η^5^-indenyl)zirconium dichloride) and **2** (isopropyliden(η^5^-3-methylcyclopentadienyl)(η^5^-fluorenyl)zirconium dichloride), and dimethylanilinium tetrakis(pentafluorophenyl)borate were obtained from TOPAS Advanced Polymers GmbH, Frankfurt, Germany. Hydrochloric acid (Sigma Aldrich; 37% *m*/*m*) was used as received.

### 2.2. Analytical Measurements

The NMR spectra were recorded on a Bruker Advance 400 instrument (400 MHz (^1^H); 100.58 MHz (^13^C); pulse angle = 12.50 ms; acquisition time = 0.94 s; delay = 16 s). The probehead was preequilibrated at a fixed temperature of 103 °C. Experiments were performed in a 10 mm NMR tube, samples were dissolved in C_2_D_2_Cl_4_ with hexamethyldisiloxane (HMDS) as internal standard.

Chemical shifts of different carbon atoms of norbornene and ethylene are reported in the literature [30]. The molar fraction of the sequences and norbornene content of some samples were determined by using a best-fitting procedure for the quantitative determination of the molar fractions of the stereosequences with a *R*^2^ > 99% [40].

Differential scanning calorimetry (DSC) analysis was performed on Mettler Toledo instrument using cyclic heating and cooling rates of 10 °C per minute. The samples of copolymers prepared at feed ratios of [N]/[E] = 26.0 and 4.8 were heated from 40 to 220 °C; samples of copolymers prepared at a feed ratio of [N]/[E] = 1.3 were heated from −50 to 220 °C. The values of glass transition temperature *T*_g_ were recorded during the second thermal cycle.

Molar mass analysis was performed using about 12 mg of polymer in *o*-dichlorobenzene at 145 °C by a GPC V2000 high temperature size exclusion chromatography (SEC) system from Waters (Millford, MA, USA), equipped with two online detectors: a viscometer (DV) and a differential refractometer (DRI). The column set was composed of three mixed TSKGel GMHXL-XT columns from Tosohaas. Calibration was conducted by using 18 narrow MWD polystyrene standards, with a molar mass ranging from 162 to 5.48 × 10^6^ g/mol.

### 2.3. Polymerization Reaction

All copolymerizations were performed in a 0.6 L stainless steel Büchi autoclave equipped with a heat jacket. The reactor was conditioned by three vacuum/N_2_ purge cycles to remove impurities before starting a polymerization. Evacuation was conducted for 60, 45 and 30 min at 85 °C to remove impurities. Catalyst solution was prepared in a glove box by dissolving precatalyst and equimolar amounts of TIBA, used for alkylation of the zirconocene dichloride compounds, in freshly distilled toluene. The solution was stirred for 3 h to guarantee complete dissolution. Then, the solid cocatalyst (dimethylanilinium tetrakis(pentafluorophenyl)borate) was introduced into the solution ([B]/[Zr] = 1.5) and the solution was stirred for an additional 45 min. The reactor was cooled to the polymerization temperature (70 °C) and charged with 150 mL toluene solution containing triisobutylaluminum, as impurity scavenger [41], and the proper amount of norbornene and diethylzinc. After thermal equilibration under mechanical stirring (about 20–30 min), the reactor was saturated with ethylene at the desired pressure. Then, catalyst solution was injected into the autoclave using a nitrogen over pressure. The ethylene pressure was kept constant during copolymerization reactions. The copolymerization time was 15 min. The copolymerization was quenched by adding some mL of ethanol into the autoclave; the polymer was precipitated in distilled ethanol and stirred overnight. The precipitated copolymer was re-dissolved in toluene and re-precipitated under mechanical stirring in pure acetone. The copolymer was filtered and dried under vacuum at 75 °C.

## 3. Results

### 3.1. Synthesis and Characterization of Poly(E-co-N)

The two catalyst precursors isopropyliden(η^5^-cyclopentadienyl)(η^5^-indenyl)zirconium dichloride (**1**) and isopropyliden(η^5^-3-methylcyclopentadienyl)(η^5^-fluorenyl)zirconium dichloride (**2**) were chosen as two typical ansa-metallocenes (Figure 1) leading to poly(ethylene-*co*-norbornene) with different microstructures. Catalyst precursors were activated by addition of dimethylanilinium tetrakis(pentafluorophenyl)borate, known to generate non-coordinating species in the activation process [40,41,42,43,44], and hence, being appropriate for the study of the diethylzinc interactions with active catalytic species, where the presence of an intimate ion pair of the cationic zirconium species with a coordinating anionic species might decrease or even inhibit the chain transfer mechanism.

All polymerization reactions were performed in the presence of both catalyst precursors in a lab-scale autoclave at 70 °C at three different [N]/[E] ratios of 1.3, 4.8 and 26.0. Polymerization conditions, similar to those used for copolymerization experiments in the presence of a single catalyst precursor [33], were chosen to assure good catalytic activities, norbornene conversions around 20 mol %. Ethylene pressure was kept constant during the polymerization and varied for the three [N]/[E] ratios (pE = 19 bar for [N]/[E] = 1.3, pE = 10 bar for [N]/[E] = 4.8 and pE = 4 bar for [N]/[E] = 26.0). Variable amounts of diethyl zinc, [Zn]/[Zr] ratios of ≈100, 200, and 400, were used to perform copolymerization reactions. In each series, the same amount of TIBA was used as alkylating agent and as scavenger [41]. Although TIBA could also be considered as a possible chain transfer agent, in E-*co*-N polymerizations with ansa-zirconocenes activated by MAO as well as in propylene polymerization by ansa metallocenes activated with TIBA and tritylborate as cocatalyst, it was shown to have low or no tendency to exchange with Zr-polymeryl chains [45,46]. Selected results of copolymerization reactions performed at [N]/[E] ratios of 1.3, 4.8 and 26.0 at 70 °C are reported in Table 1, Table 2 and Table 3, respectively. They are compared to copolymerization results obtained with single catalysts [33]. The molar ratios between the two catalysts [**1**]/[**2**] in Table 1, Table 2 and Table 3 are 1, 0.83, and 0.67, respectively. These values were selected considering the polymerization activity of the single catalysts to have polymerization activities on the two catalysts as similar as possible. All copolymers obtained were compared with their reference cases in terms of *M*_w_, polydispersity (*D*), norbornene content and *T*g_s_.

Copolymerization activities with catalyst system **1**+**2** are high and comparable to those achieved with a single catalyst. It is worth noting that norbornene content in the copolymers (entries 1, 4, and 7) at different Zn concentrations falls between those of the reference copolymers prepared by a single catalyst. This shows that both catalysts are active in the reaction environment. Norbornene content does not seem to be influenced by the variation of diethylzinc in the medium.

**Figure 2:**
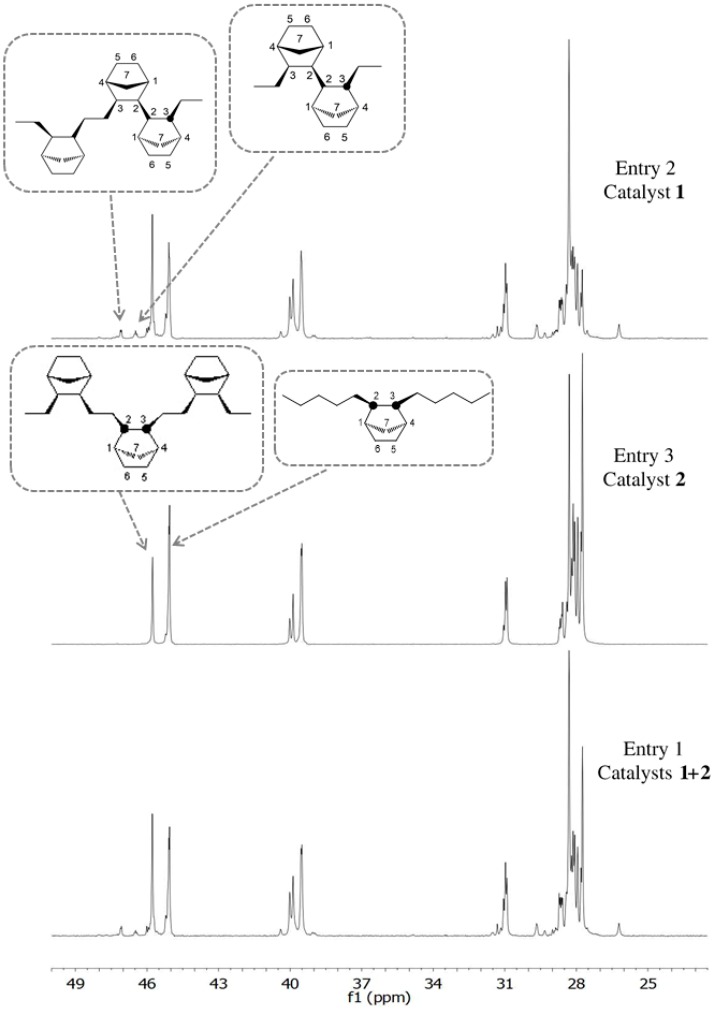
Comparison of ^13^C-NMR spectra of poly(ethylene–*co*–norbornene) samples entry 1 (prepared with catalysts **1**+**2**), entry 2 (prepared with catalyst **1**) and entry 3 (prepared with catalyst **2**) synthesized using the feed ratio [N]/[E] = 1.3.

In Figure 2, ^13^C-NMR spectra of copolymers prepared by catalysts **1** and **2** (entry 1) against those of copolymers obtained by catalyst **1** (entry 2) and catalyst **2** (entry 3) are compared. The ^13^C-NMR spectrum of the copolymer (entry 1) obtained by the binary catalytic systems is, at first sight, similar to that achieved with catalyst **1**. Indeed, this ^13^C-NMR spectrum shows peaks, assigned to NN dyads (e.g., 48.07 and 47.12 ppm due C3 carbons of ENNE *racemic* and *meso* sequences, respectively, and 26.23 and 29.68 ppm due C5 and C6 carbons, respectively, in ENNE *meso* sequences) [29], that are characteristic of copolymers synthesized by catalyst **1**.

There is only a slight decrease of *M*_w_ of copolymers obtained by raising the concentration of diethylzinc, while molecular weight distribution (*D* parameter) is quite narrow, lower than molecular weight distribution of copolymers obtained by **1** (from 1.7 to 1.9). *D* values are lower than a theoretical Schulz–Flory distribution and tend to a theoretical Poisson distribution, as it should be when diethylzinc acts according to a perfect chain transfer mechanism in a chain shuttling copolymerization [13,44]. As already reported in Ref. [33], it was not possible to obtain the *M*_w_ value of copolymers synthesized by single catalyst **2**, because of isorefractivity of these copolymers (with 21–22 mol % of norbornene content) and the SEC solvent [47,48]. The DSC thermograms of the ethylene-norbornene copolymers obtained at a [N]/[E] feed ratio of 1.3 by the presence of both catalysts do not show a well evident glass transition (vide infra). However, the registered decrease of *T*_g_ values with increasing [Zn]/[Zr] ratios reflects the decrease of *T*_g_ observed in the case of copolymers prepared by sole catalyst **1** [33]. Such an effect was considered as an indication that chain transfer to ZnEt_2_ might be reversible, and that it is more probable that it occurs after one or two inserted ethylene units [33]. Thus, this characteristic can be explained by a highly disordered microstructure of the synthesized segmented copolymers. We argue that in the copolymers obtained by both catalysts **1**+**2** the glass transition is not pronounced because in an ethylene rich environment, the chain transfer mechanism becomes more effective, inducing a shortening of the average length of the blocks as a consequence.

**Figure 3:**
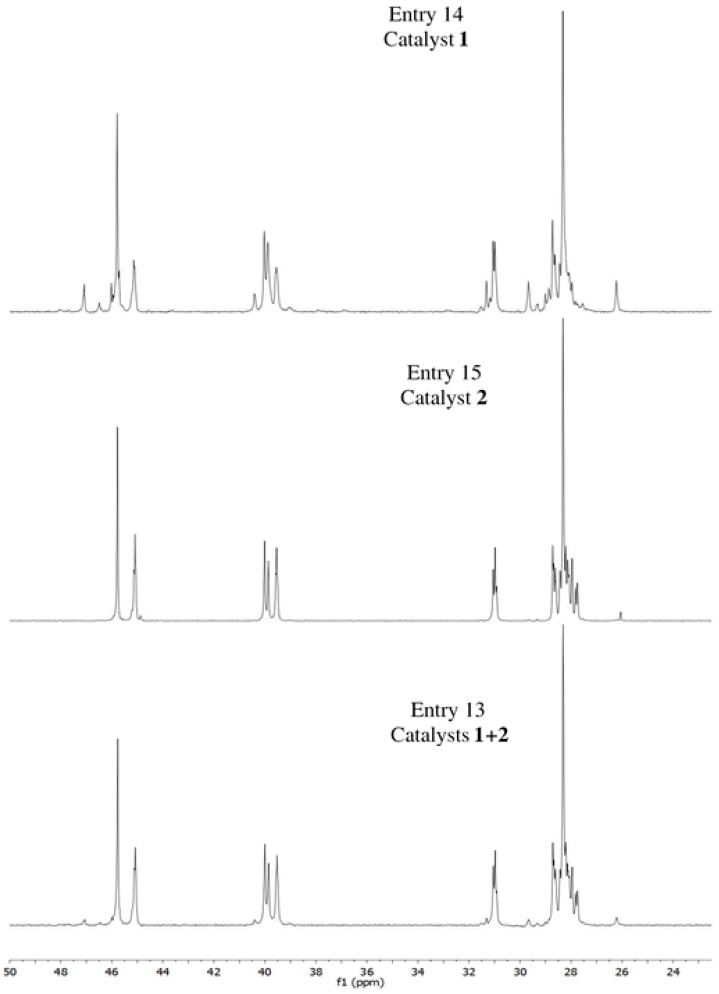
Comparison of ^13^C-NMR spectra of poly(ethylene–*co*–norbornene) samples entry 13 (prepared with catalyst **1**+**2**) against entry 14 (prepared with catalyst **1**) and entry 15 (prepared with catalyst **2**) synthesized using the feed ratio [N]/[E] = 4.8.

Copolymerization experiments performed at [N]/[E] feed ratio of 4.8 with both catalysts (entries 10, 13 and 16 in Table 2) show high catalytic activity [49]. Thus, both catalysts are active in the reaction environment. As reported in Figure 3, the ^13^C-NMR spectrum of the copolymer (entry 13) synthesized by the binary system presents clearly evident peaks, ascribed to ENNE *meso* and *racemic* sequences, characteristic of copolymers obtained by catalyst **1**, and similarity with spectrum of copolymers obtained with catalyst **2**. N content is within those of copolymers prepared by single catalysts **1** or **2**.

DSC analysis of copolymers obtained in the presence of **1**+**2** shows a single *T*_g_ at temperatures intermediate between those of reference copolymers. *M*_w_ values of copolymers obtained by the binary systems, such as those of copolymers obtained by single catalyst, become lower with increasing amounts of diethylzinc and are in between those of the reference copolymers (e.g., entry 10 vs. entries 11 and 12), as shown in Figure S1 reported in Supplementary Materials. The molecular weight distribution (*D*) is broader than in reference copolymers, but it is still compatible with homogeneous processes. The presence of a single *T*_g_, as well as an intermediate N content and *M*_w_ values, indicate the existence of homogenous copolymer chains.

A similar set of experiments was carried out at a [N]/[E] molar ratio equal to 26.0 to study the effect of diethylzinc as chain transfer agent in the presence of a high amount of N comonomer. Copolymerization reactions were performed at three [Zn]/[Zr] ratios (entries 19, 22, and 25). Copolymers obtained by dual systems have norbornene content and *T*_g_s in between those of the reference copolymers prepared by each single catalyst, with exception of entry 22, with N content and *T*_g_ value above that of entry 23. Diethylzinc reduces the *M*_w_ of copolymers synthesized in the presence of the two catalysts, while giving quite narrow molecular weight distributions, very close to the Schulz–Flory distribution.

*M*_w_s are lower than *M*_w_ reported for copolymers obtained by catalyst **1** (which is the catalyst that produces lower *M*_w_ copolymers). This may be due to the high norbornene content that reduces the rate of propagation of the catalytic system, especially when the copolymer chain is bound to catalyst **2**, and the transfer between the two catalytic systems occurs.

### 3.2. Chain End Analysis and Number of Chains

**Figure 4:**
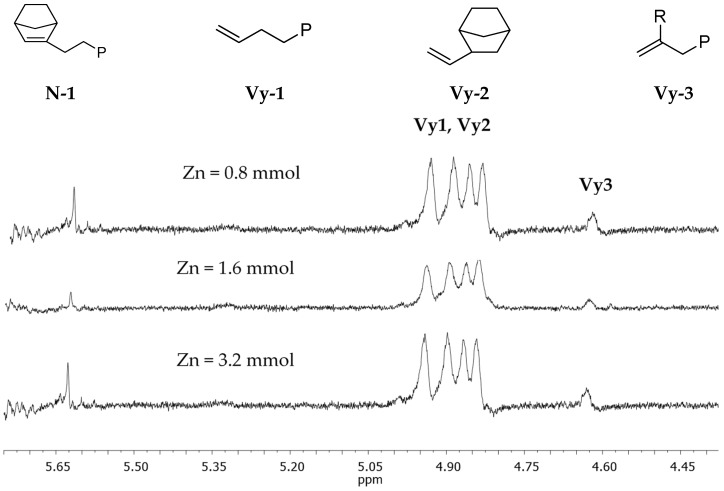
Expansions of the region between 4.4 and 5.75 ppm of ^1^H NMR spectra (400 MHz, C_2_D_2_Cl_4_, 103 °C) of poly(E-*co*-N)s prepared by **1**+**2** at [N]/[E] feed ratio of 1.3 and different amount ZnEt_2_ (Table 1): (**top**) [Zn]/[Zr] = 55 (entry 1); (**middle**) [Zn]/[Zr] = 99 (entry 4); and (**bottom**) [Zn]/[Zr] = 198 (entry 7).

The analysis of copolymer chain ends showed that the characteristic chain transfers of E-*co*-N polymerization take place in addition to chain transfers to zinc alkyl [33]. The spectra of copolymers obtained by **1** showed the existence of vinyl linked to norbornene (Vy-2) or ethylene (Vy-1) units and vinylidenes rising when a vinyl terminated polymer chain (Vy-3) is reinserted, and β-H elimination follows. Norbornenyl end group (N-1), a singlet at 5.62 ppm, becomes the prevailing one in spectra of copolymers with high N content (see Figure S2 in Supplementary Materials). The ^1^H NMR spectra of copolymers synthesized by **2** showed the multiplets of vinylidene groups (Vy-1 and Vy-2) and some signals of internal double bonds arising from isomerization at 5.20 and 5.35 ppm. When increasing the norbornene content, molar masses are higher and signals of terminal groups are less visible (see Figure S3 in Supplementary Materials). Addition of diethyl zinc to both systems did not change the terminal groups visibly. The ^1^H NMR spectra of copolymers prepared in the presence of the two catalysts show the chain end groups typical of the two catalytic systems (see Figure 4), where the ^1^H NMR spectra of entries 4, 7 and 9, prepared in the presence of different [Zn]/[Zr] ratios are reported.

The total number of terminal groups per 10,000 monomer insertions is reported in Table 1 along with copolymer chain numbers per catalytic center for copolymers synthesized at [N]/[E] feed ratio of 1.3, where the values are more accurate compared to those at higher feed ratios. The chain number for catalytic center, calculated from yield/(*M*_n__*_ cat center), indicates the number of polymer chains grown per each catalyst molecule. As already reported in Ref. [33], the number of copolymer chains obtained by catalyst **1** is always much higher than those produced by catalyst **2**. This is related to lower molar masses of copolymers synthesized by **1**. It lessens with increasing [N]/[E] ratios, as the copolymer chains are richer in norbornene and molar masses increase since chain transfer and β–H elimination are more facile after one or two last inserted ethylene units. What was instructive was the comparison of polymer chains and end groups produced in the presence of different amounts of diethyl zinc in the series obtained at a [N]/[E] ratio of 1.3 with catalyst **1**, which provided more accurate values. Implications of possible reaction mechanism were discussed. Here, we only note that in copolymers synthesized at a [N]/[E] feed ratio of 1.3 with the two catalysts, the number of polymer chains per catalyst is similar to those obtained by **1**, while the number of terminal groups is more similar to those obtained by **2**. By increasing the [N]/[E] feed ratios, the number of polymer chains lessens and the values are within those of the two single catalysts.

### 3.3. Microstructure

**Scheme 2:**
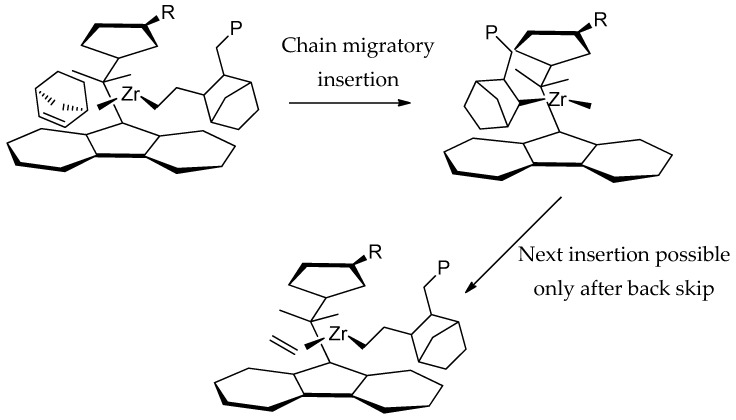
Copolymerization mechanism for E-N copolymerization by C1 symmetric metallocenes of type *i*-Pr[(3-R-Cp)(Flu)]-ZrCl_2_.

High-resolution ^13^C NMR is one of the most valuable methods for studying copolymer composition and microstructure. Some of us have devoted significant efforts in elucidating the complex ^13^C NMR spectra of poly(E-*co*-N)s at tetrad or even pentad level in the past. This has allowed for a more precise test of the statistical model best describing E-*co*-N copolymerization and the polymerization mechanism. The influence of ligand substitution of methylaluminoxane activated *C*_1_ symmetric catalysts *i*-Pr-[(3-R-Cp)(Flu)]ZrCl_2_ (R = Me or *i*-Pr) was studied and the statistical model best describing E-*co*-N copolymerization was tested [40]. It was found that norbornene and ethylene are inserted at the same site by a Cossee migratory mechanism, and then the copolymer chain backskips to its original position after every insertion (Scheme 2).

The synthesis of alternating poly(E-*co*-N)s, possible only at very high [N]/[E] feed ratios, originates from the impossibility of having two consecutive norbornene insertions. The isotacticity derives from the norbornene insertion being always at the same site with the same face. Penultimate (second-order Markov) effects play a crucial role especially in E-*co*-N copolymerization with *i*-Pr[(3-*i*-Pr-Cp)(Flu)]ZrCl_2_ (**3**).

We applied this methodology at tetrad level to analyze the copolymers obtained by single catalytic systems in the presence and in absence of CTA as well as those obtained in the presence of the dual catalytic system in the presence of different amounst of CTA. It is known that, when the insertion of a comonomer is affected by the last inserted unit (ultimate effect) or by the penultimate unit, a first- or a second-Markovian statistical model is used to determine the reactivity ratios [50].

In our studies on E-*co*-N copolymerization, we found that, when bulky monomers such as norbornene are engaged, second-order models are often required to describe the copolymerization.

From the first-order Markovian model, the *r*_1_ (=*k*_11_/*k*_12_) and *r*_2_ (=*k*_22_/*k*_21_) reactivity ratios are determined, where *k_ij_* is the rate constant of the reaction for the addition of the monomer *j* to a growing chain having the comonomer *i* as the last inserted unit. Here, 1 and 2 indicate ethylene and norbornene, respectively.

When the second-order Markov statistical model is necessary to describe the copolymerization, four reactivity ratios are defined:

*r*_11_ = *k*_111_/*k*_112_ = *k*_EEE/_*k*_EEN_   and  *r*_22_ = *k*_222_/*k*_221_ = *k*_NNN/_*k_NNE_*

*r*_21_ = *k*_211_/*k*_212_ = *k*_NEE/_*k*_NEN_   and  *r*_12_ = *k*_122_/*k*_121_ = *k*_ENN/_*k*_ENE,_

where *k*_lmn_ represents the rate constant for the insertion of monomer *n* into an *lm*-metal ending chain.

Both first- and second-order statistical models were used to fit the experimental tetrad distributions of copolymers synthesized by the **1**+**2** dual catalytic system in the presence of different amount of ZnEt_2_. Some selected results of series performed at [N]/[E] ratios of 1.3 and 4.8 are reported in Table S1.

**Figure 5:**
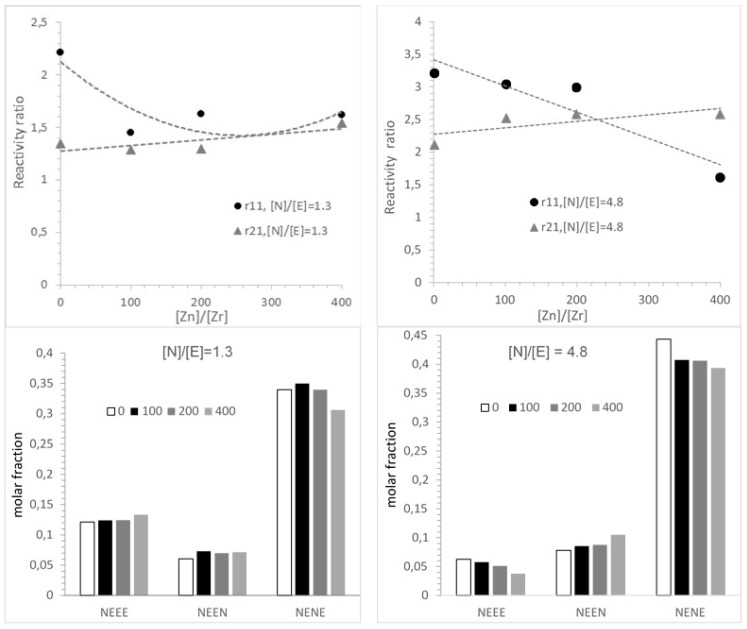
Comparison of microstructural analysis of poly(E-*co*-N)s obtained with catalyst **1** at 1.3 and 4.8 [N]/[E] feed ratios in the presence of increasing amount of ZnEt*_2_*: (**top**) *r*_11_ and *r*_21_ values (A); and (**bottom**) NEEE, NEEN and NENE tetrad molar fractions (B).

All series follow the second-order Markov statistics. The main differences between **1** and **2** catalytic systems are in the *r*_11_ and *r*_21_ values. Both *r*_11_ and *r*_21_ values of poly(E-*co*-N)s obtained with catalyst **2** are higher than those of poly(E-*co*-N)s obtained with catalyst **1**, which indicates that norbornene insertion is more difficult with catalyst **2** than with catalyst **1**. Catalyst **1** has rather open sites, while **2** follows the mechanism mentioned above. Differences are greater in poly(E-*co*-N)s synthesized at higher [N]/[E] feed ratios. Adding or increasing ZnEt_2_ at [N]/[E] feed ratios of 1.3 results in *r*_11_ values of copolymers which change slightly and tend to decrease, while *r*_21_ values increase slightly. At [N]/[E] feed ratios of 4.8, the trend is similar. This is evident in copolymerization reactions with catalyst **1** (see Figure 5). The decrease of *r*_11_ values indicates that, on average, by raising the amount of CTA with catalyst **1**, there is a decrease of the preference to insert a third ethylene unit after two consecutive ethylene insertions, and the increase of *r*_21_ values indicates an increase of the preference to insert a second ethylene unit in Mt-EN bond. This is also clear from a comparison of NEEE, NEEN and NENE tetrad molar fractions as a function of CTA, as shown in Figure 5B.

When we used the catalytic systems together in the presence of different amounts of ZnEt_2_, the values are between those of the single catalysts, but they are more similar to those of copolymers obtained by catalyst **1**. This is an indication that the copolymer chains grow more on catalyst **1** than on catalyst **2.**

### 3.4. Analysis of the Structural, Thermal and Mechanical Properties

Significant information on structure and properties of some ethylene/1-octene multiblock copolymers produced by chain shuttling technology were achieved by analysis of the structural, thermal, and mechanical properties [22,23,24]. This analysis also gave relevant information on the effect of ZnEt_2_ on the chain microstructure of poly(E-*co*-N) samples prepared with the sole catalysts **1** and **2** [33]. This analysis revealed that the samples obtained with catalyst **1** are amorphous regardless of the amount of CTA used in the polymerization. The samples prepared at [N]/[E] feed ratios of 4.8 with catalyst **2** are also amorphous, whereas the samples prepared at [N]/[E] feed ratios of 1.3 and 26 with catalyst **2** present some crystallinity. In particular, the copolymers obtained at [N]/[E] feed ratio of 1.3 show crystallization of long ethylene sequences in the orthorhombic form of polyethylene (PE), whereas the copolymers obtained at [N]/[E] feed ratio of 26 show crystallinity from long alternating NENE sequences in the crystalline form of the poly(E-*co*-N) isotactic alternating copolymers [51,52]. DSC analysis revealed a melting temperature of 125–130 °C in the case of the samples obtained at [N]/[E] feed ratio of 1.3, and of 260–280 °C in the case of the samples obtained at [N]/[E] feed ratio of 26, regardless of CTA concentration, indicating that the average length of crystallizable sequences is not affected by CTA.

**Figure 6:**
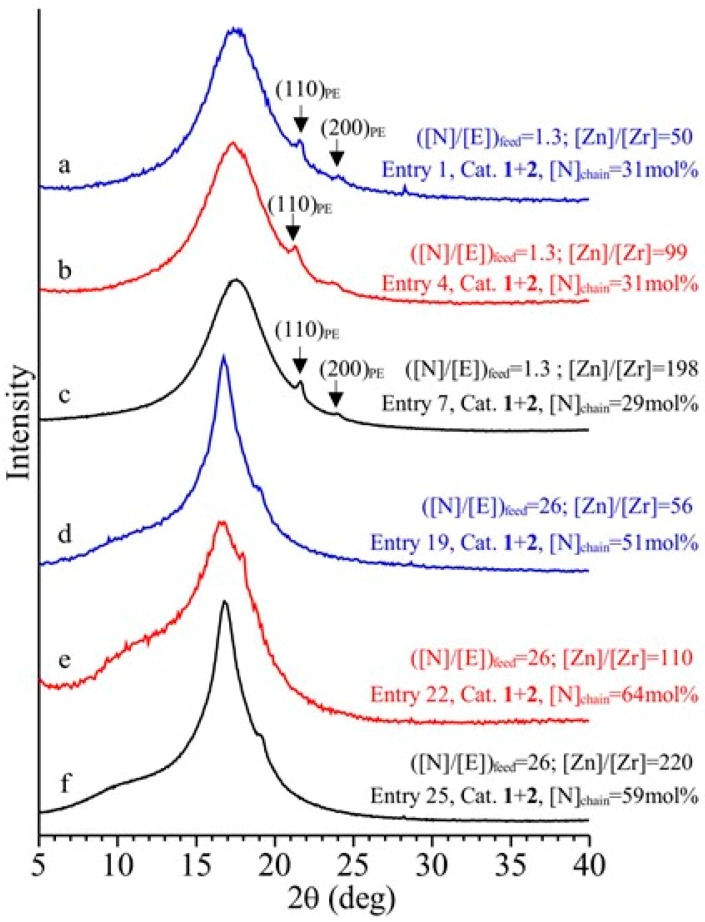
X-ray powder diffraction profiles of melt crystallized poly(ethylene-*co*-norbornene) samples obtained with catalysts **1**+**2** at [N]/[E] feed ratio of 1.3, 4.8 and 26.0, using the indicated values of the [Zn]/[Zr] ratio during polymerization.

The X-ray powder diffraction profiles and DSC thermograms of the poly(E-*co*-N)s obtained with the mixture of the two catalysts **1**+**2** and different concentrations of CTA are shown in Figure 6 and Figure S2, respectively. It is apparent that the X-ray diffraction profiles of the samples prepared at [N]/[E] feed ratio of 1.3 (Figure 6, curves a–c) show diffraction peaks at 2θ ≈ 21 and 24°, indicating that also these samples contain ethylene sequences that crystallize in the orthorhombic form of PE, in agreement with results of Ref. [33] On the other hand, the X-ray diffraction profiles of the samples prepared at [N]/[E] feed ratio of 26 (Figure 6, curves d–f), show diffraction peaks at 2θ ≈ 17 and 19°, due to the crystallization of alternating EN sequences, in analogy with the samples prepared with the sole catalyst **2** [33]. Finally, the diffraction profiles of the samples prepared at [N]/[E] feed ratio of 4.8 (data not shown), indicate that these samples are amorphous, in analogy with the samples prepared with the catalysts **1** and **2** separately.

The melting temperatures of the polyethylene-like crystals of ≈125 °C (Figure S2, curves a–c) and of the crystals of the alternating EN copolymer of ≈270 °C (Figure S2, curves g–h) in samples obtained with the mixture of catalysts **1** and **2**, are similar to those formed in the samples obtained with the sole catalyst **2**. This indicates that the average length of crystallizable sequences is not affected by the CTA concentration, even using both catalysts **1**+**2**. Moreover, similar to the copolymers obtained in presence of the sole catalyst **2**, when using a [N]/[E] feed ratio of 26, the DSC thermograms of the poly(E-*co*-N)s copolymers obtained by the two catalysts **1**+**2** also show cold crystallization at ≈200 °C in the second heating scan, indicating that these samples do not crystallize from the melt during the heating scan at 10 °C/min (data not shown).

**Figure 7:**
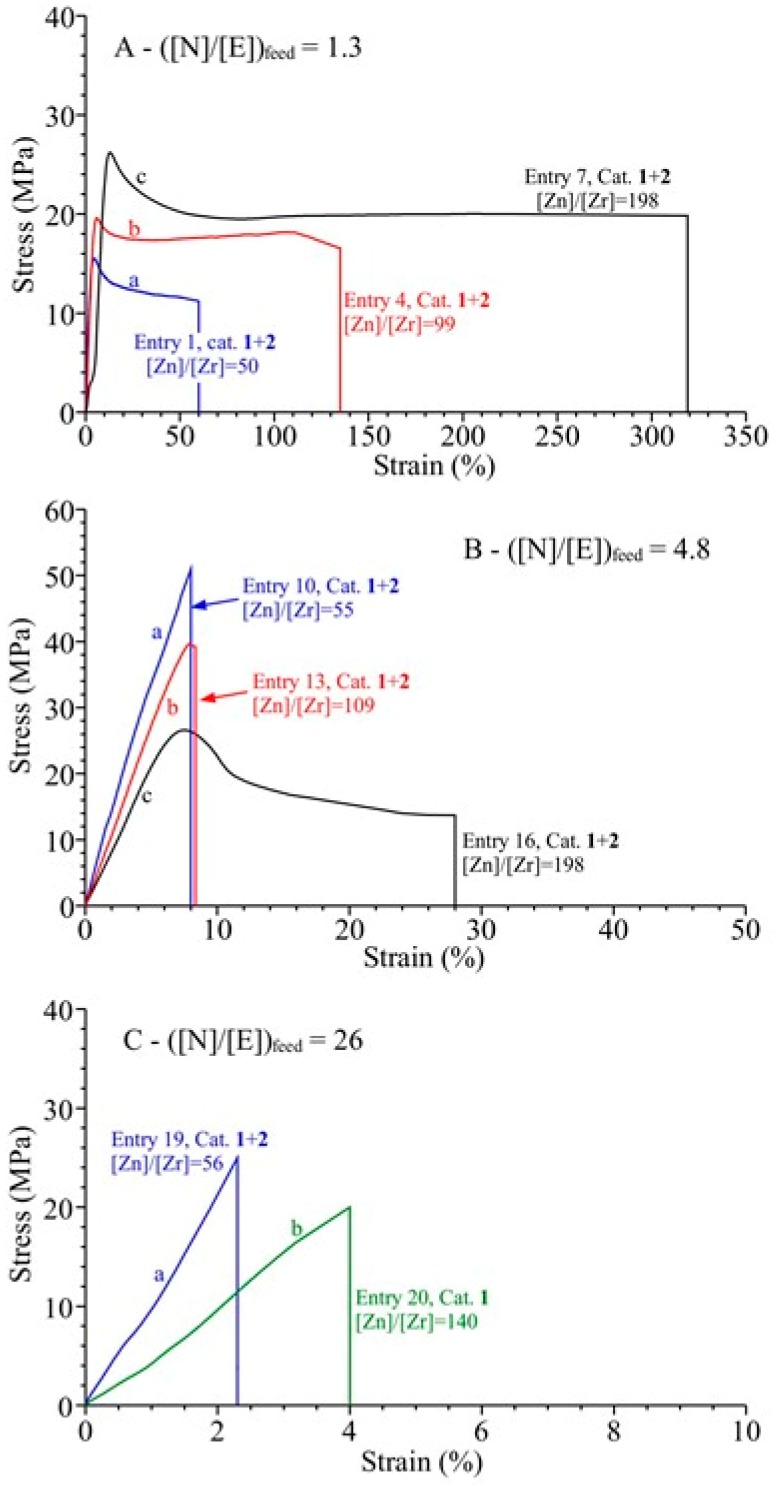
Stress–strain curves of compression molded films of poly(ethylene-*co*-norbornene) copolymers synthesized with the catalysts **1**, **2** and **1**+**2**, at [N]/[E] feed ratio of: 1.3 (**A**); 4.8 (**B**); and 26 (**C**). The concentration of CTA used in the polymerization is indicated as [Zn]/[Zr] ratio. All samples were stretched at rate *v* = 0.5 *l_0_* with *l_0_* the initial gauge length, with exception of entry 7 in (**A**), which was stretched at rate *v* = 10 *l*_0_.

The stress–stain tensile curves of the poly(E-*co*-N) samples obtained by the two catalysts **1**+**2** at [N]/[E] feed ratio of 1.3, 4.8 and 26 are reported in Figure 7. The leading mechanical parameters extracted from stress–strain curves are reported in Table S2.

In general, whereas the samples prepared at [N]/[E] feed ratio of 4.8 and 26 are rigid and fragile, and break at very low deformations (Figure 7B,C), the samples prepared at [N]/[E] feed ratio of 1.3 are more flexible and show much higher ductility (Figure 7A). In particular, in the case of the samples prepared at [N]/[E] feed ratio of 4.8 with both catalysts **1**+**2** (entries 10, 13, and 16), the Young’s modulus and the stress at any strain tend to decrease, whereas the deformation at break tends to increase with increasing the amount of CTA (Figure 7B and Table S2).

Moreover, whereas entries 10 and 13 break before yielding at deformation lower than 10%, as in the case of the corresponding copolymers obtained with the sole catalysts **1** and **2** [33], entry 16, obtained at higher CTA concentration, shows diffuse yielding and deformation at break of ≈28%. Therefore, due to CTA, at [N]/[E] feed ratio of 4.8, amorphous copolymers are produced, characterized at least in part by a segmented chain architecture, generated by the chain transfer mechanism from **1** to **1**, from **2** to **2** and from **1** to **2** or **2** to **1**, respectively. The flexibility and ductility increase with increasing CTA concentration, probably because the frequency of exchange also increases, and the segments produced in consecutive turnovers are possibly jointed through flexible ethylene sequences.

A high fragility and elevated rigidity also characterize the samples obtained at [N]/[E] feed ratio of 26 (Figure 7C and Table S2). The values of Young’s modulus reaches vales of 600–900 MPa, and the deformation at break is lower than 4–5% (Table S2). In particular, as analyzed in Ref. [33], the samples produced with catalyst **2** break immediately by application of a tensile force, because the crystals formed by long NE alternating sequences act as reinforcing elements of the glassy matrix, resulting in a neat increase of rigidity, but also increase of fragility. On the contrary, the samples produced with catalyst **1** show a slightly higher ductility, with values of deformation at break and mechanical strength that tend to increase with increasing CTA concentration [33]. In Figure 7C, the stress–strain curves of entry 20, produced with the catalyst **1** at [Zn]/[Zr] feed ratio of 140, with N content of 64 mol%, and entry 19, produced with the two catalysts **1**+**2** at [Zn]/[Zr] feed ratio of 56, with N content of 51 mol%, are compared. It is apparent that the two samples show similar ductility, but entry 19 shows a higher value of the Young’s modulus (900 MPa, Table S2) and higher mechanical strength than entry 20 (Figure 7C), despite the lower norbornene concentration. This reinforcing effect is due not only to the presence of the crystals formed by the long NE alternating sequences, but also to the blocky chain architecture of entry 19, consisting of rigid segments produced by catalyst **1**, covalently linked to the crystallizable NENE alternating sequences produced by catalyst **2**. The copolymers obtained by both catalysts **1**+**2**, entry 22 and 25 at higher [Zn]/[Zr] concentration, are even more rigid, and break at even lower deformations than entry 19 (data not shown). The increase of rigidity is probably due to the fact that, with the increase of the CTA concentration, the exchange frequency also increases, and in a high norbornene environment the chain transfer mechanism to catalyst **1** is more probable. Therefore, whereas the chain transfer mechanism produces a softening effect in the case of the amorphous samples produced by the sole catalyst **1**, in the case of the semi-crystalline samples obtained wit catalyst **2**, or with the mixture **1**+**2**, a reinforcing effect occurs, due to the crystals formed by the alternating NE sequences produced by **2**, and/or by the increased amount of norbornene rich segments produced by **1.**

The Young’s modulus was determined in independent experiments at *v*/*L*_0_ = 0.1mm/(min mm). The stress–strain behavior of poly(E-*co*-N)s obtained at [N]/[E] feed ratio of 1.3 with catalysts **1**+**2** (entries 1, 4 and 7), prepared in presence of increasing amount of CTA (Figure 7A), clearly confirms that these samples, at least in part, by effect of the CTA mechanism, possess a segmented chain architecture, with segments produced by catalysts **1** and **2** alternating along the chain. In fact, since the molecular mass of these samples remains constant, the observed decrease of the Young’s modulus and increase of the mechanical strength and ductility with increasing CTA concentration are a direct consequence of the increase of the exchange frequency of the growing chains between the catalysts, leading not only to a decrease of the average length of the polymer segments produced in consecutive turnovers at the metallic centers, but also to an increase of junction points and their average number for each chain. In particular, the decrease of the average length of segments produced by **2** may account for the decrease of Young’s modulus, whereas the increase of the average number of segments/chain produced by the two catalysts, and especially of the more rigid segments produced by the more efficient catalyst **1**, may account for the increase of mechanical strength and ductility.

**Figure 8:**
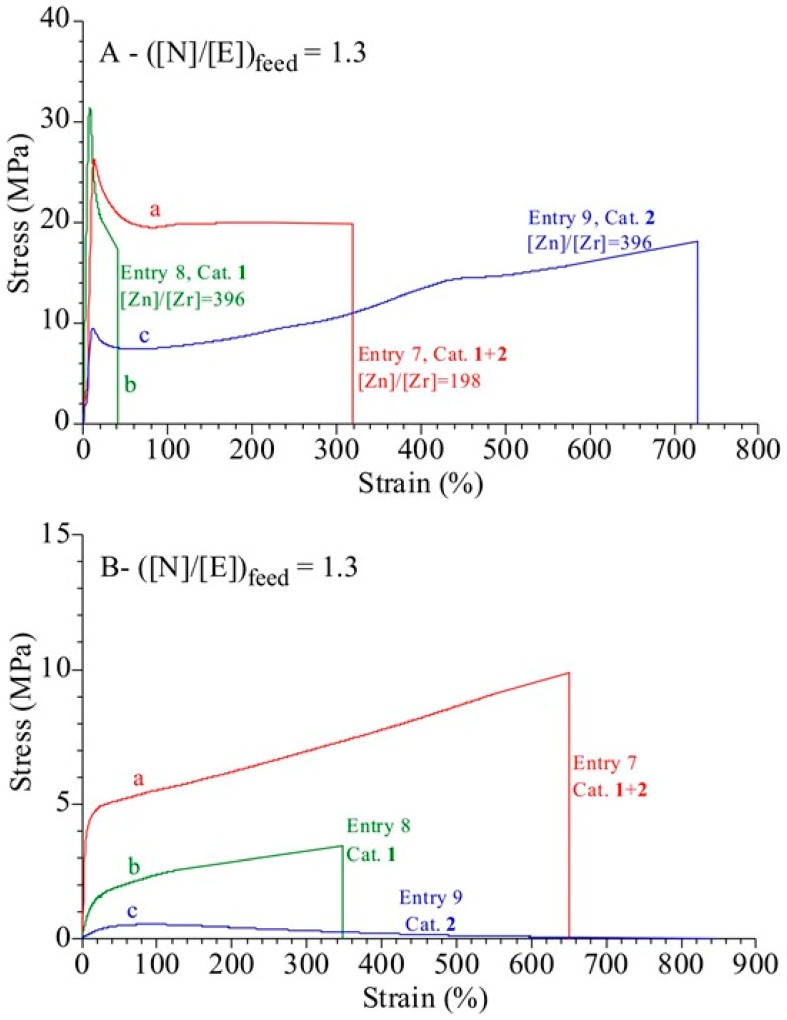
Stress–strain curves of compression molded films of poly(ethylene-*co*-norbornene) copolymers synthesized with the catalysts **1**, **2** and **1**+**2**, at [N]/[E] feed ratio of 1.3 and at the highest [Zn]/[Zr] ratio (entries 7, 8 and 9), stretched at: 25 °C (**A**); and 50 °C (**B**). All samples were stretched at rate *v* = 10 *l*_0_ with *l*_0_ the initial gauge length.

**Figure 9:**
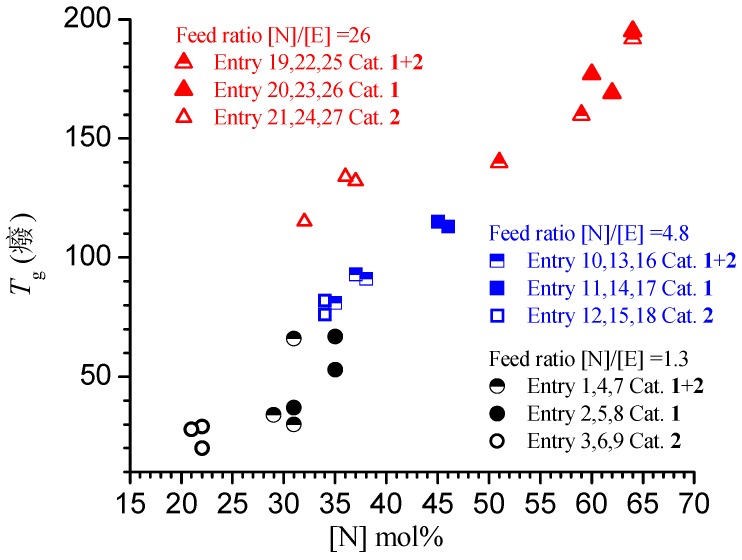
Glass transition temperatures (*T*_g_) evaluated from the DSC heating thermograms of samples cooled from high temperature of poly(ethylene-*co*-norbornene) obtained with catalysts **1** (full symbols), **2** (empty symbols) and catalysts **1**+**2** (half full symbols) at [N]/[E] feed ratio of 1.3 (triangles), 4.8 (square) and 26.0 (triangles) and catalysts.

Further information concerning the effect of CTA in the production of multiblock copolymers may be obtained by comparing the mechanical behavior recorded at 25 °C and at temperatures higher than the glass transition temperature, for triplets of samples produced using a high CTA concentration, with the sole catalyst **1**, the sole catalyst **2** and with the mixture **1**+**2**. As an example, the stress strain curves of entries 7, 8 and 9 recorded at 25 and 50 °C are shown in Figure 8. These samples are obtained at [N]/[E] feed ratio of 1.3, using the highest CTA concentration and show glass transition temperatures of 34, 37 and 20 °C, respectively (Table 1 and Figure 9).

As discussed in Ref. [33], at 25 °C (Figure 8A) entry 8, obtained with the sole catalyst **1** shows a high value of the Young’s modulus (280 MPa), high stress at yield (32 MPa), but low deformation at break (41%). Entry 9, obtained with the sole catalyst **2**, shows low Young’s modulus (53 MPa) and low stress at yield (10 MPa), but high deformation at break (700%). Entry 7, obtained with the catalysts **1**+**2**, shows values of Young’s modulus (170 MPa) and stress at yield (26 MPa), similar to those of entry 8, but ductility intermediate between those of entries 8 and 9 (deformation at break of 300%). The properties of entry 7, obtained with the catalysts **1**+**2**, are not those expected for a simple blend using entries 8 and 9 as components in the appropriate amount, but strongly support the hypothesis that, by effect of CTA, in presence of the catalysts **1**+**2**, a reactor blend of chains is obtained, consisting of different fractions produced not only by the chain transfer from **1** to **1**, and from **2** to **2**, but also by a fraction of segmented chains produced by exchange from **2** to **1** and **1** to **2,** which act as compatibilizer.

Concerning the mechanical tests performed at 50 °C, (Figure 8B), it is apparent that, compared with the stress–strain curves at room temperature of Figure 8A, the amorphous sample produced by **1** (entry 8), becomes much softer at 50 °C (Figure 8B, curve b).

The Young’s modulus and stress at break drop to ≈2 MPa and 3.5 MPa, respectively, while the deformation at break increases to 350% (Figure 8, curve b). The semi-crystalline sample produced by **2** (entry 9) shows viscous flow by stretching, already starting from 100% deformation (Figure 8, curve c) and the value of the Young’s modulus drops to 0.9 MPa. Finally, the sample obtained with the mixture of catalysts **1**+**2** (entry 7) shows outstanding mechanical properties even at 50 °C. The deformation at break increases up to the value of 650%, the Young’s modulus is reduced to 43 MPa, whereas the stress at break is still high at 10 MPa (Figure 8, curve a). Moreover, a partial elastic recovery of the initial dimension occurs, with value of tension at break of 215%. Therefore, whereas this sample (entry 7) experiences plastic deformation at room temperature, it becomes partially elastic at 50 °C. The mechanical properties of entry 7 at 50 °C are clearly different from those expected by a blend of the parent samples entries 8 and 9. This is the hallmark that this sample is characterized, at least in part, by multiblock chains consisting of norbornene rich (produced by **1**) and norbornene poor (produced by **2**) segments, alternating along the chain.

## 4. Conclusions

The effect of variable amounts of ZnEt_2_ as chain transfer agent on the synthesis of poly(E-*co*-N)s in the presence of two catalyst precursors **1** and **2**, was evaluated to verify the occurrence of a chain transfer mechanism. Copolymerization experiments were performed at elevated polymerization pressure and high temperatures, applying three different concentrations of norbornene in the feed. N contents, *M*_w_ and *T*_g_ values of copolymers prepared in the presence of precatalysts **1**+**2** are intermediate between those of reference copolymers. From chain end group analysis, it was observed that the number of polymer chains per catalyst is more similar to those obtained by **1**, while the number of terminal groups is more similar to those obtained by **2**. Microstructural analysis revealed that all series follow the second-order Markov statistics. The main difference between copolymers synthesized by **1** and **2** catalytic systems is in the *r*_11_ and *r*_21_ values. Both *r*_11_ and *r*_21_ values of poly(E-*co*-N)s obtained with catalyst **2** are higher than those of poly(E-*co*-N)s obtained with catalyst **1**. Differences are greater in poly(E-*co*-N)s synthesized at higher [N]/[E] feed ratios. When copolymers are obtained by the two catalytic systems together in the presence of different amounts of ZnEt_2_, the values of *r*_11_ and *r*_21_ are between those of copolymers prepared by single catalysts, but they are more similar to those of copolymers obtained by catalyst **1**. This result, along with the number of polymer chains and chain end groups, is an indication that the copolymer chains grow more on catalyst **1** than on catalyst **2.**

Interestingly, the study of tensile and elastic properties of poly(E-*co*-N)s prepared by **1**+**2** in the presence of different amounts of CTA, gives evidence that copolymers were obtained through exchange of polymer chains between different transition metal sites. Indeed, the change of ductility, rigidity, and mechanical strength with increase of CTA in the polymerization reaction, was the result of the segmented microstructure of the chains, produced through a chain transfer mechanism between zirconium and zinc.

A significant insight on the effect of CTA is obtained by analyzing the mechanical properties of the copolymers prepared at [N]/[E] feed ratio of 1.3, that is, in conditions where chain exchanges are more probable [33]. In particular, for three samples obtained at the highest [Zn]/[Zr] ratio, the similar values of Young’s modulus and stress at yield for the copolymers obtained with the mixture of catalysts **1**+**2** and those obtained with the catalyst **1**, and the fact that the values of ductility for the copolymers obtained with **1**+**2** are intermediate between those of the reference copolymers, may be taken as a clear indication of CTA efficiency. These properties, indeed, indicate that, in the presence of CTA, the copolymers obtained with the mixture **1**+**2** possibly consist of a reactor blend of segmented chains produced not only by the chain transfer from **1** to **1**, and from **2** to **2**, but also include segments produced by exchange from **2** to **1** and **1** to **2** acting as an ideal compatibilizer. Furthermore, in the case of poly(ethylene-*co*-norbornene) obtained at [N]/[E] feed ratio of 1.3 with the catalysts **1**+**2**, the observed decrease of the Young’s modulus with the increase of the CTA concentration may be ascribed to the increase of junction points between the segments, possibly occurring in correspondence to (flexible) ethylene sequences. On the other hand, for these systems, since the number of rigid segments produced by **1** covalently linked to crystallizable segments produced by **2** increases with increase of CTA concentration, the mechanical strength and ductility also increase. Therefore, the mechanical behavior of these samples is the result of the segmented chain architecture produced by the two catalysts **1**+**2** in presence of CTA.

The efficiency of CTA in presence of catalysts **1**+**2** in the production of multiblock copolymers including a fraction of chains consisting of amorphous blocks synthesized by **1** alternating with blocks synthesized by **2**, is also confirmed by testing their mechanical properties at temperatures higher than the glass transition. The sample obtained with the mixture **1**+**2** shows outstanding mechanical properties even at 50 °C. Moreover, whereas the samples experience plastic deformation at room temperature, the multiblock copolymers obtained by **1**+**2** present elastomeric behavior at 50 °C. The mechanical properties of these samples at 50 °C are clearly different from those expected by a blend of the reference samples prepared with the two separate catalysts. This is the hallmark that these samples contain a fraction of multiblock chains consisting of norbornene rich (produced by **1**) and norbornene poor (produced by **2**) segments, alternating along the chain.

## Figures and Tables

**Table 1:** Poly(Ethylene–*co*–Norbornene) using catalysts **1** and **2** in the presence of ZnEt_2_ at [N]/[E] feed ratio of 1.3 and pE = 19 bar.

Entry ^a^	Catalyst	Zn (mmol)	[Zn]/[Zr]	Yield (g)	Activity ^b^	N (mol %) ^c^	Conv (%)	*T*_g_ (°C) ^d^	*M*_w_ × 10^−3^ (Kg/mol) ^e^	*D* ^e^	Chain Number for Catalytic Center ^f^	Chain End per 10,000 units ^g^
1	**1+2**	0.8	50	18.4	242	31	42	66	27	1.8	77	8.6
2	**1**	0.8	99	9.3	244	35	23	67	30	1.9	72	14.1
3	**2**	0.8	99	9.7	255	22	18	29	---	---	--	4
4	**1+2**	1.6	99	16.8	221	31	36	30	25	1.8	77	6.2
5	**1**	1.6	198	7.2	189	35	17.5	53	29	1.9	59	13
6	**2**	1.6	198	9.5	250	21	17.1	28	---	---	---	3.8
7	**1+2**	3.2	198	8.4	110	29	18	34	23	1.7	39	8.7
8	**1**	3.2	396	5.3	139	31	12	37	29	1.8	41	11.8
9	**2**	3.2	396	9.8	258	22	18	20	---	---	---	3.6

^a^ Polymerization conditions: Temperature = 70 °C; time = 15 min; Zr = 8 μmol (1), 8 μmol (2); dimethylanilinium tetrakis(pentafluorophenyl)borate [B]/[Zr] = 1.5; TIBA = 8 mmol; ^b^ Activity is expressed as kg(polymer)/(mol(cat) × h × bar); ^c^ Norbornene incorporation is obtained by ^13^C-NMR spectroscopy; ^d^ *T*_g_ is obtained by DSC; ^e^ Molecular masses and polydispersity D = *M*_w_/*M*_n_ are obtained by SEC; ^f^ Number of polymer chains calculated from Yield/(*M*_n_ × cat center); ^g^ Number of Chain Ends per 10,000 units calculated from integral of unsaturated chain ends in ^1^H NMR spectra by taking into account the composition of the copolymer chain.

**Table 2:** Poly(Ethylene–*co*–Norbornene) using catalysts **1** and **2** in the presence of ZnEt_2_ at [N]/[E] feed ratio of 4.8 and pE = 10 bar.

Entry ^a^	Catalyst	Zn (mmol)	[Zn]/[Zr]	Yield (g)	Activity ^b^	N (mol %) ^c^	Conv (%)	*T*_g_ (°C) ^d^	*M*_w_ × 10^−3^ (Kg/mol) ^e^	*D* ^e^	Chain Number for Catalytic Center ^f^
10	**1+2**	1.2	55	16	290	37	20.6	93	239	2.9	9
11	**1**	1	100	1.7	68	46	2.4	113	61	1.9	5
12	**2**	1.2	100	5.9	197	34	7.2	82	309	1.7	3
13	**1+2**	2.4	109	15.9	289	38	21	91	155	2.7	13
14	**1**	2	200	11	440	45	16	115	64	2	34
15	**2**	2.4	200	1.5	50	34	1.8	76	170	2.9	2
16	**1+2**	4.8	198	9.7	176	35	12	81	113	2.9	11
17	**1**	4	400	0.7	28	46	1	113	48	2	3
18	**2**	4.8	400	3.9	130	34	4.8	76	97	2.6	9

^a^ Polymerization conditions: Temperature = 70 °C; time = 15 min; Zr = 10 μmol (**1**), 12 μmol (2); dimethylanilinium tetrakis(pentafluorophenyl)borate [B]/[Zr] = 1.5; TIBA = 15 mmol; ^b^ Activity is expressed as kg(polymer)/(mol(cat) × h × bar); ^c^ Norbornene incorporation is obtained by ^13^C-NMR spectroscopy; ^d^ *T*_g_ is obtained by DSC; ^e^ Molecular masses and polydispersity *D* = *M*_w_/*M*_n_ are obtained by SEC; ^f^ Number of polymer chains calculated from Yield/(*M*_n_ × cat center).

**Table 3:** Poly(Ethylene–*co*–Norbornene) using catalysts **1** and **2** in the presence of ZnEt_2_ at [N]/[E] feed ratio of 26.0 and pE = 4 bar.

Entry ^a^	Catalyst	Zn (mmol)	[Zn]/[Zr]	Yield (g)	Activity ^b^	N (mol %) ^c^	Conv (%)	*T*_g_ (°C) ^d^	*M*_w_ × 10^−3^ (Kg/mol) ^e^	*D* ^e^	Chain Number for Catalytic Center ^f^
19	**1+2**	2.8	56	7.9	158	51	5.5	140	99	2.2	4
20	**1**	2.8	140	15.8	790	64	12.1	195	146	5.4	13
21	**2**	2.8	93	1	33	37	0.6	132	---	---	---
22	**1+2**	5.5	110	21.6	432	64	16.6	192	53	1.9	16
23	**1**	5.5	275	1.7	85	60	1.3	177	54	2	3
24	**2**	5.5	183	2	67	36	1.2	134	122	2	1
25	**1+2**	11	220	8.8	176	59	6.5	160	27	2	13
26	**1**	11	550	0.2	10	62	0.2	169	40	3.1	1
27	**2**	11	367	0.4	13	32	0.2	115	77	4.8	1

^a^ Polymerization conditions: Temperature = 70 °C; time = 15 min; Zr = 20 μmol (1), 30 μmol (2); dimethylanilinium tetrakis(pentafluorophenyl)borate [B]/[Zr] = 1.5; TIBA = 20 mmol; ^b^ Activity is expressed as kg(polymer)/(mol(cat) × h × bar); ^c^ Norbornene incorporation is obtained by ^13^C-NMR spectroscopy; ^d^ *T*_g_ is obtained by DSC; ^e^ Molecular masses and polydispersity *D* = *M*_w_/*M*_n_ are obtained by SEC; ^f^ Number of polymer chains calculated from Yield/(*M*_n_ × cat center).

## Supplementary Materials

The following are available online at https://www.mdpi.com/2073-4360/11/3/554/s1, (1) Details of structural, thermal and mechanical characterization; (2) Figure S1 Molecular mass distribution for the sample obtained at for [N]/[E = 4.8 and Zn = 1,2 mmol; (3) Figure S2 ^1^H NMR of spectra of copolymers prepared by each catalyst with and without ZnEt_2_; (4) Figure S2 Expansions of the region between 4.4 and 5.75 ppm of ^1^H NMR spectra of polymers prepared by **2**; (5) Figure S4 DSC thermograms recorded during the II heating scan of melt crystallized poly(ethylene-*co*-norbornene) samples obtained with catalysts **1**+**2**; (6) Table S1 Reactivity Ratios of E-*co*-N Copolymerization Reactions; (7) Table S2 Values of the Young’s modulus (E), stress and strain at yield (ε_y_ and σ_y_) and at break (ε_b_ and σ_b_), and tension set at break (t_b_).
