# Supplementary material for: Ethylene-co-norbornene Copolymerization Using a Dual Catalyst System in the Presence of a Chain Transfer Agent
Source: Polymers (Basel). 2019 Mar 22;11(3):554. doi: 10.3390/polym11030554 (PMC6473246; doi:10.3390/polym11030554)
Supplement: Supplementary file 1 [file polymers-11-00554-s001.pdf]

Supporting Information:

Ethylene–*co*–norbornene Copolymerization Using a Dual Catalyst System in the Presence of a Chain Transfer Agent

Laura Boggioni,[1] Diego Sidari,[1] Simona Losio,[1] Udo M. Stehling,[2]† Finizia Auriemma,[3]* Anna Malafronte, [3] Rocco Di Girolamo, [3] Claudio De Rosa,[3]and Incoronata Tritto[1]*

[1] Istituto per lo Studio delle Macromolecole (ISMAC), Consiglio Nazionale delle Ricerche (CNR), Via E. Bassini 15, 20133 Milano, Italy;

[2] TOPAS Advanced Polymers GmbH, Paulistrasse 3, 65926 Frankfurt am Main, Germany

[3] Dipartimento di Scienze Chimiche, Università di Napoli Federico II, Complesso Monte S. Angelo, Via Cintia, 80126 Napoli, Italy;

* Correspondence: E-mail: tritto@ismac.cnr.it, auriemma@unina.it

†SABIC Technology Center Riyadh, P.O. Box 42503, Riyadh 11551, Kingdom of Saudi Arabia

PREPARATION OF COMPRESSION MOLDED FILMS

Compression molded films were prepared by heating the as polymerized samples at temperatures 20-30 °C higher than the melting temperatures under a press. They were successively cooled to room temperature at average rate of ≈10°C/min. The applied pressure was kept low, in order to avoid preferred orientations in the samples.

X-RAY ANALYSIS

Wide angle X-ray scattering (WAXS) data were collected on compression-molded films using the multipurpose diffractometer Empyrean (PANalytical) in the θ-θ reflection geometry, with CuKα◎incident ◎radiation (wavelength λ =0.15418 nm).

THERMAL ANALYSIS

DSC thermograms were obtained with a differential scanning calorimeter Mettler Toledo DSC-1 performing scans in a flowing N2 atmosphere and heating or cooling at a rate of 10°C/min.

MECHANICAL TESTS

The mechanical tests were performed at room temperature on unoriented compression molded films using universal mechanical tester Instron, following the standard test method for tensile properties of thin plastic sheeting ASTM D882-83. Rectangular specimens 10 mm long, 5 mm wide and 0.3 mm thick were cut from compression molded films and stretched up to the break. Two benchmarks were placed on the test specimens and used to measure elongation. In the mechanical tests the ratio between the drawing rate and the initial length was fixed equal to 0.1 mm/(mm×min) for the measurement of Young's modulus, and 10 or 0.5 mm/(mm×min), depending on the rigidity, of the sample, for the measure of the stress-strain curves up to break. The reported curves and values of Young modulus were averaged over at least five independent experiments.

Values of tension set were measured on unoriented compression molded films after breaking. Two benchmarks were drawn on the specimens at distance L_0. Then the samples were stretched up to the break, i.e. up to achieve elongation $\varepsilon_b = [(L_f - L_0)/ L_0]100$, where Lf is the distance between the benchmarks at breaking. Ten minutes after breaking the two pieces of the sample were fit carefully together so that they are in contact over the full area of the break and the final total length Lr of the specimen was obtained by measuring the distance between two benchmarks. The tension set t_b after breaking was calculated as: $t_b = [(L\text{r} - L_0)/ L_0]\times 100$.

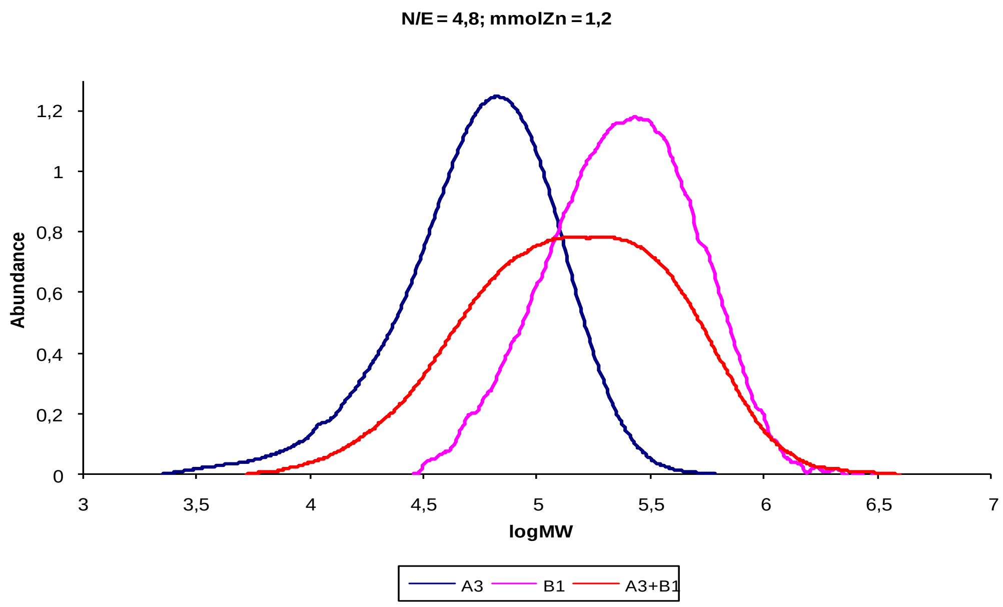

Figure S1. Molecular weight profiles for [N]/[E] = 4.8 and Zn = 1,2 mmol (A3 = 1; B1= 2)

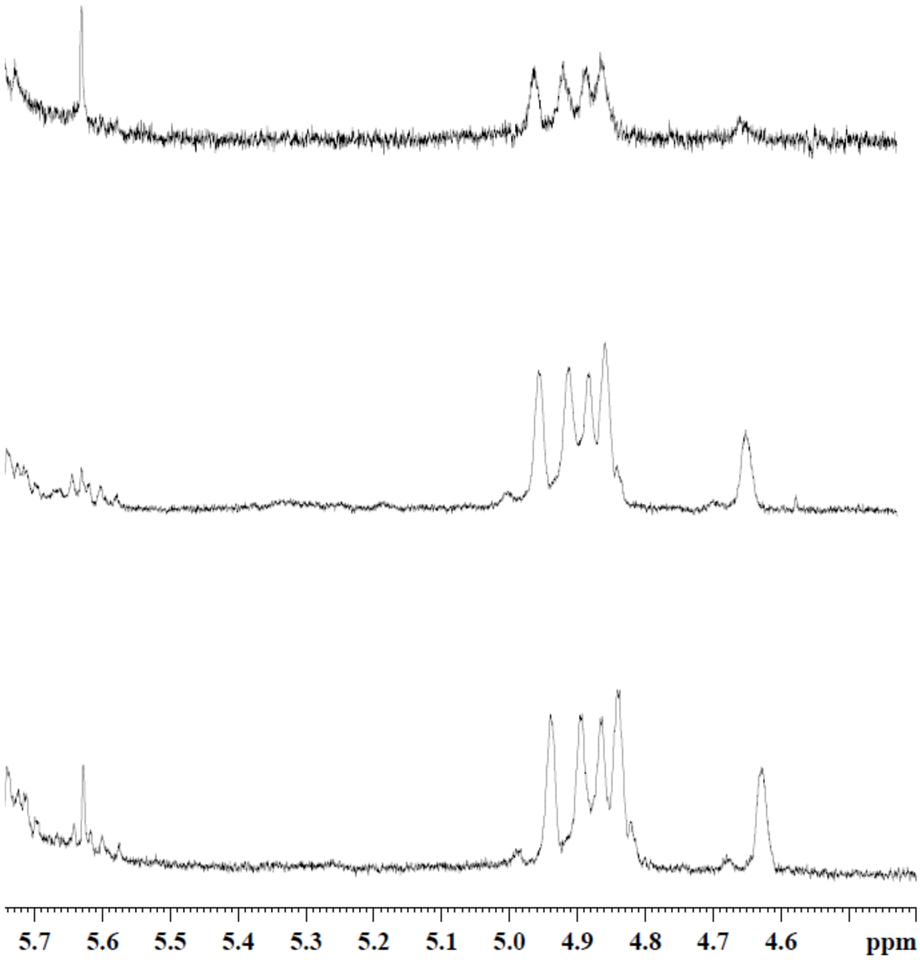

Figure S2. Expansions of the region between 4.4 and 5.75 ppm of ^{1}H NMR spectra (400 MHz, $C_2D_2Cl_4$, 103 °C) of polymers prepared by **1**:

a) (top): poly(E-co-N) without $ZnEt_2$ at [N]/[E] feed ratio of 4.8 (Table 3, entry 3 in ref. [33];

b) (middle): poly(E-*co*-N) without $ZnEt_2$ at [N]/[E] feed ratio of 1.3 (Table 1, entry 1 in ref. [33];

c) (bottom): poly(E-*co*-N) with $ZnEt_2$ at [N]/[E] feed ratio of 1.3 (Table 1, entry 8).

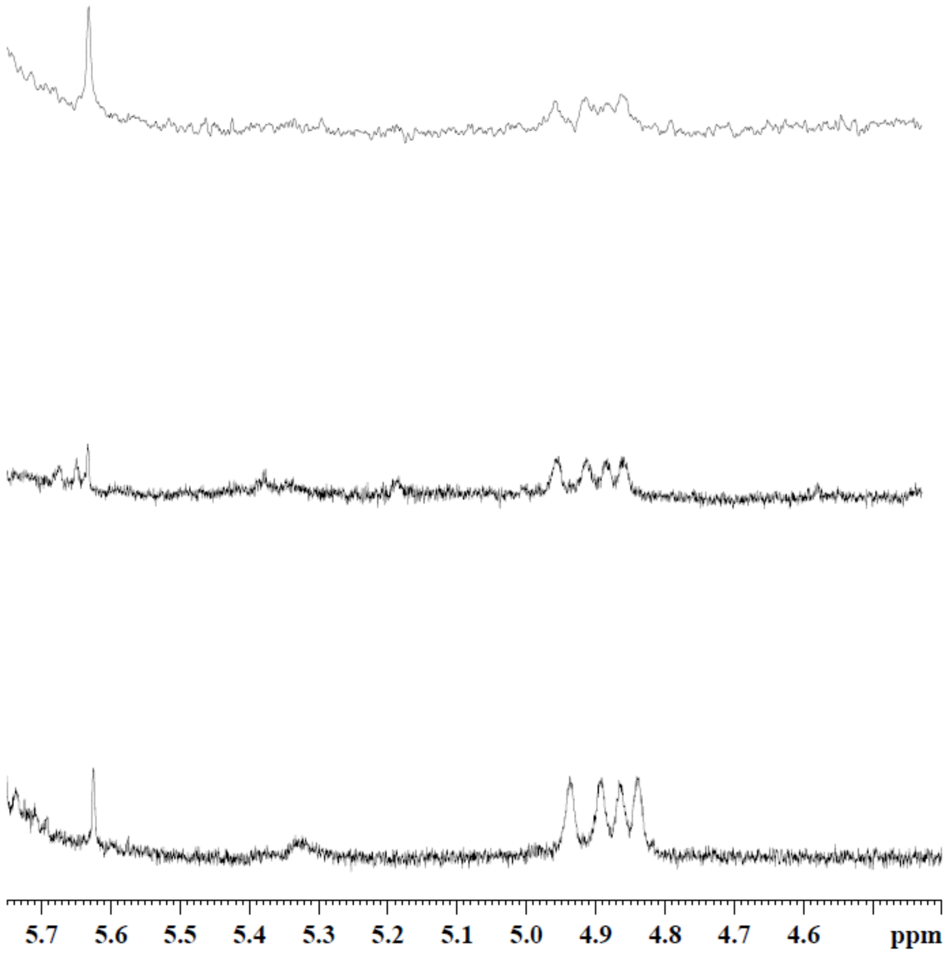

Figure S3. Expansions of the region between 4.4 and 5.75 ppm of ^{1}H NMR spectra (400 MHz, C2D2Cl4, 103 °C) of polymers prepared by **2:**
a) (top): poly(E-*co*-N) without $ZnEt_2$ at [N]/[E] feed ratio of 4.8 (Table 3, entry 4 in ref. [33]);
b) (middle): poly(E-*co*-N) without $ZnEt_2$ at [N]/[E] feed ratio of 1.3 (Table 1 entry 2 in ref [33]);
c) (bottom): poly(E-*co*-N) with $ZnEt_2$ at [N]/[E] feed ratio of 1.3 (Table 1, entry 9)

^{1}H NMR spectra of copolymers obtained show the signals of end groups, essentially terminal vinyl groups bonded to inserted ethylene or norbornene units, depending on the N content in the copolymer.

As reported in ref. [33] by adding diethyl zinc to both systems there is no change in the terminal groups visible.

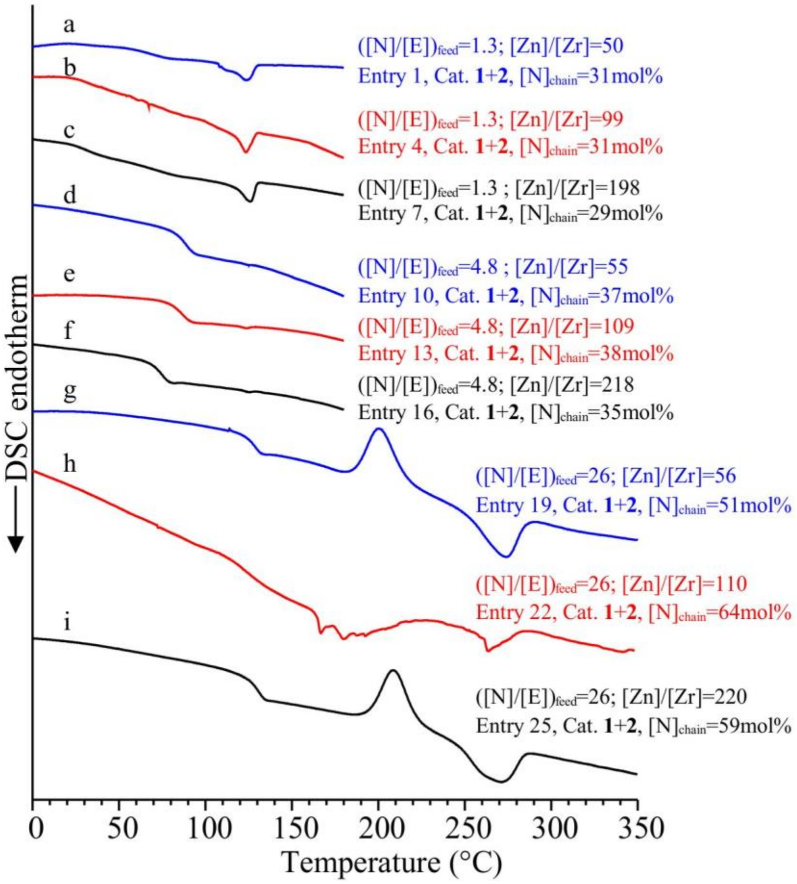

Figure S4. DSC thermograms recorded during the II heating scan of melt crystallized poly(ethylene-co-norbornene) samples obtained with catalysts **1**+ **2** at [N]/[E] feed ratio of 1.3, 4.8 and 26.0, using the indicated values of the [Zn]/[Zr] ratio during polymerization.

Table S1 Reactivity Ratios of E-*co*-N Copolymerization Reactions with Catalyst **1**, **2** and **1+2** Calculated using First and Second Order Markovian Models from Tetrad Distribution

cat	[N]/[E]	[Zn]/[Zr]	N mol %[a]	r_1	r_2	r_{11}	r_{12}	r_{21}	r_{22}
**1**	1.3	0	35	1.65	0.03	2.21	0.01	1.35	0.00
**1**	1.3	100	35	1.34	0.01	1.45	0.01	1.29	0.00
**1**	1.3	200	35	1.40	0.02	1.63	0.02	1.30	0.07
**1**	1.3	400	31	1.57	0.03	1.62	0.03	1.54	0.13
**2**	1.3	0	20	3.76	0.00	4.27	0.00	2.21	0.00
**2**	1.3	100	22	3.26	0.00	3.81	0.00	1.98	0.00
**2**	1.3	200	21	3.48	0.00	4.08	0.00	1.98	0.00
**2**	1.3	400	22	1.57	0.03	1.62	0.03	1.54	0.00
**1+2**	1.3	50	31	1.69	0.03	2.65	0.01	1.21	0.30
**1+2**	1.3	99	31	2.06	0.03	2.89	0.02	1.40	0.00
**1+2**	1.3	198	29	1.88	0.04	2.52	0.02	1.42	0.00
**1**	4.8	0	45	2.23	0.05	3.21	0.05	2.11	0.00
**1**	4.8	100	46	2.60	0.05	3.04	0.06	2.52	0.00
**1**	4.8	200	45	2.63	0.05	2.99	0.06	2.58	0.00
**1**	4.8	400	46	2.42	0.05	1.61	0.06	2.58	0.00
**2**	4.8	0	33	5.49	0.00	6.75	0.00	4.92	0.02
**2**	4.8	100	34	3.27	0.00	4.62	0.00	3.04	0.00
**2**	4.8	200	34	4.70	0.01	5.10	0.01	4.56	0.00
**2**	4.8	400	34	-	-	5.15	0.02	3.46	0.00
**1+2**	4.8	55	37	2.89	0.02	3.53	0.02	2.78	0.00
**1+2**	4.8	100	38	3.34	0.03	4.69	0.03	3.08	0.00
**1+2**	4.8	200	35	3.46	0.02	5.74	0.02	3.02	0.00

[a] Determined from tetrads

Table S2 Values of the Young modulus (E), stress and strain at yield (ε_y, σ_y) and at break (ε_b, σ_b), and tension set at break (t_b) extracted from the stress strain curves of Figure 7 for the poly(ethylene-*co*-norbornene) copolymers, obtained by catalyst **1** and/or **2**, at different concentrations of $ZnEt_2$ as CTA.[a]

Entry	E	N	Catalyst	[Zn]/[Zr]	Zn mmol	M_w (10^{-3})	v/L_0 (min-	E (MPa)	σ_b(MPa)	ε_b(%)	σ_y (MPa)	ε_y(%)	t_b(%)
**([N]/[E])$_{feed}$ =1.3**													
1	69	31	**1+2**	50	0.8	27	0.5	370±40	11±1	60±10	15.7±0.5	4.8±0.6	-
3	78	22	**2**	99	0.8	-	10	300±20	37±3	740±70	15.1±0.7	9±2	-
4	69	31	**1+2**	99	1.6	25	0.5	300±100	17±2	135±20	20±3	6±1	-
5	65	35	**1**	198	1.6	29	0.5/10	310±50	13±1/14±2	70±20/40±20	20±2/24±4	12±2/13±1	-
6	79	21	**2**	198	1.6	-	10	240±80	36±3	900±100	20±2	10.7±0.8	490±70
7	71	29	**1+2**	198	3.2	23	10	170±50	20±1	320±90	26±2	13±1	300±100
8	69	31	**1**	396	3.2	29	0.5/10	280±20	19±1/17±4	110±20/41±9	26±2/32±7	12.5±0.5/12.0±0.3	-
9	78	22	**2**	396	3.2	-	10	53±6	18±3	700±100	10±1	11.0±0.5	300±30
**([N]/[E])$_{feed}$ =4.8**													
10	63	37	**1+2**	55	1.2	239	0.5	650±50	51±6	8±1	-	-	-
13	62	38	**1+2**	109	2.4	155	0.5	600±90	39±6	8.3±1.3	-	-	-
16	65	35	**1+2**	198	4.8	113	0.5	470±80	14±2	24±9	27±4	7.5±0.7	-
**([N]/[E])$_{feed}$ =26**													
19	49	51	**1+2**	56	2.8	99	0.5	900±100	25±4	2.3±0.5	-	-	-
20	36	64	**1**	140	2.8	146	0.1	600±100	20±4	4±1	-	-	-
23	40	60	**1**	275	5.5	54	0.5	500±100	13±3	5±2	-	-	-

a) The samples have been stretched at indicated values of deformation rate v/L

“The authors declare no conflict of interest.” Authors must identify and declare any personal circumstances or interest that may be perceived as inappropriately influencing the representation or interpretation of reported research results. Any role of the funders in the design of the study; in the collection, analyses or interpretation of data; in the writing of the manuscript, or in the decision to publish the results must be declared in this section. If there is no role, please state “The funders had no role in the design of the study; in the collection, analyses, or interpretation of data; in the writing of the manuscript, or in the decision to publish the results”.

© 2019 by the authors. Submitted for possible open access publication under the terms and conditions of the Creative Commons Attribution (CC BY) license (http://creativecommons.org/licenses/by/4.0/).
